# Attitudes of care staff towards video consultations

**DOI:** 10.1192/bjb.2020.91

**Published:** 2020-10

**Authors:** Rory Shadwell, Colin Hemmings, Max Pickard

**Affiliations:** Foundation Year 2 doctor , East Kent Hospitals University NHS Foundation Trust, UK; email: rory.shadwell@nhs.net; Consultant Psychiatrist in Intellectual Disability, Kent and Medway NHS and Social Care Partnership Trust, UK; Consultant Psychiatrist in Intellectual Disability, Kent and Medway NHS and Social Care Partnership Trust, UK

We read with interest the excellent article by Johns et al on video consultations in mental health services.^1^ Such video consultations can also be used with key informants such as care staff. In our specialist mental health service for people with an intellectual disability (also known in UK health services as learning disability) in Kent we have just completed a quality improvement project on attitudes towards video consultations among the staff in care settings.

We found that the majority of the care staff interviewed felt that video consultations would not have a negative impact on access to (67%) or on the quality of care (69%) provided by our mental health service for people with intellectual disability. Additionally, we asked care staff if they would consider using video consultations in place of face-to-face consultations beyond the time frame of the coronavirus disease 2019 (COVID-19) pandemic. Again, we found that the majority (66.7%) said they would.

Around a third of care staff stated that video consultations could be a good alternative to face-to-face appointments as they would allow them to still go ahead even if the service users declined to leave their accommodation. Other care staff explained that video consultations would allow clinicians to see the service users in their own environment and that they may make it easier to involve multiple healthcare professionals in an appointment. The most frequently cited benefit of video consultations was the potential to alleviate the worry and anxiety that some service users experience when going to a clinic appointment.

The attitudes towards video consultations among care staff were overall positive but they were not uniformly so. For example, it was mentioned that having a video consultation may mean that the service user is more likely to become distracted. Another respondent mentioned that for their service users, much of the information needed is derived from non-verbal communication and observed behaviour, which may be more difficult to assess over video. One carer stated that it would be too difficult to get their service user to cooperate with using the communication device.

These findings may be of particular significance in the world we face post-COVID-19 lock-down where individuals may experience increased anxiety associated with healthcare settings. The month of April 2020 saw a 48% fall in attendances to accident and emergency departments when compared with the previous year, and the fall was 72% for minor injury units and urgent care centres.^2^ There may be some long-lasting public fear surrounding healthcare settings that disproportionately affects the most vulnerable patients and telepsychiatry may prove critical in reaching those individuals as well as the staff who care for them.
